# X-ray and UV Radiation Damage of dsDNA/Protein Complexes

**DOI:** 10.3390/molecules26113132

**Published:** 2021-05-24

**Authors:** Paweł Wityk, Dorota Kostrzewa-Nowak, Beata Krawczyk, Michał Michalik, Robert Nowak

**Affiliations:** 1 Faculty of Chemistry, Gdańsk University of Technology, Narutowicza 11/12, 80-233 Gdańsk, Poland; beata.krawczyk@pg.edu.pl; 2 Faculty of Chemistry, University of Gdańsk, Wita Stwosza 63, 80-308 Gdańsk, Poland; 3 Department of Biopharmaceutics and Pharmacodynamics, Medical University of Gdańsk, Al. Gen. J. Halera 107, 80-416 Gdańsk, Poland; 4 Centre for Human Structural and Functional Research, Institute of Physical Culture Sciences, University of Szczecin, 17C Narutowicza St., 70-240 Szczecin, Poland; dorota.kostrzewa-nowak@usz.edu.pl (D.K.-N.); robert.nowak@usz.edu.pl (R.N.); 5 MML Medical Centre, Bagno 2, 00-112 Warsaw, Poland; michalim@mml.com.pl

**Keywords:** radiotherapy, photodynamic therapy, DNA damage, DNA/protein interactions

## Abstract

**Simple Summary:**

One of the most common diseases in the world is cancer. The development of an appropriate treatment pathway for cancer patients seems to be crucial to fight this disease. Therefore, solving the problem that affects more and more people in an aging society is crucial. The study presents the results of radiation and photochemical damage to DNA interacting with proteins (specifically/non-specifically). The obtained results of the analysis of photoliths and radiolites by means of the LC-MS technique allowed to identify possible mechanisms of degradation of DNA interacting with proteins. Results suggest the protective action of protein against hydroxyl radicals or solvated electrons and increased damaging effect when sensitized DNA is irradiated by UV light (280 or 320 nm) compared to the DNA alone (without protein interaction).

**Abstract:**

Radiation and photodynamic therapies are used for cancer treatment by targeting DNA. However, efficiency is limited due to physico-chemical processes and the insensitivity of native nucleobases to damage. Thus, incorporation of radio- and photosensitizers into these therapies should increase both efficacy and the yield of DNA damage. To date, studies of sensitization processes have been performed on simple model systems, e.g., buffered solutions of dsDNA or sensitizers alone. To fully understand the sensitization processes and to be able to develop new efficient sensitizers in the future, well established model systems are necessary. In the cell environment, DNA tightly interacts with proteins and incorporating this interaction is necessary to fully understand the DNA sensitization process. In this work, we used dsDNA/protein complexes labeled with photo- and radiosensitizers and investigated degradation pathways using LC-MS and HPLC after X-ray or UV radiation.

## 1. Introduction

Ionizing radiation used during cancer radiotherapy can trigger both reversible and irreversible cell DNA damage [1]. Primary DNA damage, created directly by high energy radiation, produces single or clustered damage [2,3,4]. Secondary damage also occurs when reactive oxygen species, mostly generated by radiolysis, interact with the DNA molecule [5,6]. Hydroxyl radicals and solvated electrons are the most abundant species present in these reactions [7,8]. In oxygenated cells, hydroxyl radicals are produced in higher concentrations compared to solvated electrons. However, in hypoxic conditions, solvated electrons appear at the same concentration as hydroxyl radicals [9,10]. This observation is similar to the hypoxic conditions observed in solid tumor cells with insufficient angiogenesis [11]. Despite abundance of different highly reactive radiolitic species, for native DNA, hydroxyl radicals are able to trigger more damage than solvated electrons [12].

In order to promote damage by solvated electrons it is important to use sensitizers—modified nucleotides with high electron affinity—to capture electrons from the solution for interaction with the DNA molecule. The most common, extensively studied radiosensitizers are BrdU, BrdG, BrdA, and BrdC, which are characterized by high electron affinity (HEA) and the ability to go through the dissociative electron attachment (DEA) process [13]. The DEA process leads to the dissociation of the Br- anion generating a reactive nucleobase radical which triggers DNA damage. These radiosensitizers can also employed as photosensitizers. The absorption maximum of BrdU, BrdA, BrdG, and BrdC is 285, 252, 276, and 283 nm, respectively, indicating that the use of UV light in the range of >280 nm should, to a great extent, excite the brominated analogs of nucleobases rather than native nucleobases, since the maximum of absorption of native nucleobases is around 260 nm. Photosensitizer molecules (especially bromine derivatives of nucleobases) in the excited state exhibit strong electron acceptor properties. Such properties, in combination with guanosine (G, the most easily oxidized base) can result in photoinduced DNA damage. It has been previously described that the excitation of BrdU can lead to photoinduced electron transfer between BrdU and G in the DNA molecule [14,15]. It is also worth noting that the excitation of 5-iodo-2′-deoxyuridine, can lead to homolytic cleavage of the C–I bond in contrast to the cleavage of C–Br bond in BrdU, where homolytic cleavage is less probable [16]. In addition, UV light exposure can produce the common photolesions like cyclopyrimidine dimers (CPD) and the 6-4 photoproduct (64P), 8-oxoG [17,18,19,20]. However, those lesions can be reversed by enzymatic repair in the cell. In order to create more severe and permanent DNA damage, photosensitizers are desirable to trigger DNA single or double stranded breaks [13].

Lesions induced with the use of photosensitizers often require distinct processes to occur—photoinduced electron transfer (PET) or homolysis of C–Br bond resulting in a free radical on a DNA nucleobase [21]. PET is commonly observed between the electron donor G and electron acceptor, the excited state of the photosensitizer, and the “excited” pair can produce DNA lesions. It was shown that the most sensitive sequence to the PET process is 5′-GAABrU-3′ and sensitivity is highly dependent upon the DNA sequence between the electron donor and the acceptor [22]. It was also shown previously, that electron transfer can occur between the amines present in the surrounding BrdU molecule in DNA, thus we should consider all distinct molecules present in the DNA environment [23].

Many groups are working with nucleosides, nucleotides, and DNA in aqueous solutions, which could be contaminated by different compounds and interfere with the experiments. Additionally, water solutions are missing an important factor—DNA/protein interactions [13,22,24]. In the cell environment, DNA is always interacting with many specific proteins, e.g., histones, polymerases, etc. Among the specific interaction we can distinguish the, e.g., ARG/G, ASP/A, VAL/T, GLU/C pairs [24,25]. These interactions could be very important for DNA damage, because of the change of the ionic properties of the radio- or photosensitizers in their environment [4,26,27,28]. However, in order to fully understand radiation and photo-damage to DNA, sufficiently simple systems for analysis should be employed.

In this work we would like to show the novel approach to DNA damage investigation. As indicated above, in the literature we are lacking simple enough systems to monitor DNA damage at molecular level, thus we have filled the void by constructing the dsDNA/protein system when we have all DNA/protein interaction conserved. In this work we will present the comparison of damage between dsDNA and dsDNA-protein complex due to UV or X-ray radiation. The DNA/protein complex consists of the basic fragment of GCN4 protein (common transcriptional factor) and represents the most abundant protein family (α-helix/B-DNA interaction). The main goal of the project was to determine the effect of interacting proteins in generating photochemical and X-ray radiation damage in native and sensitized DNA. In the cell environment, native DNA continuously interacts with proteins, such as transcription factors, histones, polymerases, repair enzymes, etc. Specific interactions mainly consist of hydrogen bonds between the side chains of amino acids and the nucleic bases that make up the DNA. These interactions affect the electron affinity of the nucleobases and the mechanism of damage in the sensitized DNA. Therefore, it seems necessary to expand upon research conducted thus far on photo- and radio-degradation in models of sensitized DNA assembled into peptide complexes. Investigating the efficiency of the damage process in complexes and comparing it with the degradation in isolated, point-labeled oligonucleotides, should enable characterization of the effect of amino acids/nucleobase interactions. The mechanisms of photo- and radio-degradation have also been previously described using hybrid computational methods to estimate the thermodynamic-kinetic barriers of the elementary reactions that result in oligonucleotide damage [29].

## 2. Results

### 2.1. Molecular System Description

The final goal of the work was to understand and quantify DNA sensitization in systems like those found in living cells. This paper focuses on specific interactions of a basic system consisting of a GCN4 protein fragment (bZIP) and dsDNA recognized by this peptide, described previously by Wityk et al. [30]. In order to simplify such a complicated process, a model system was made in which a fragment of bZIP (25 aa) is covalently connected to a 17 bp dsDNA oligonucleotide by the CLICK reaction. The obtained complex was characterized by Differential Scanning Calorimetry (nano-DSC), circular dichroism (CD), High Performance Liquid Chromatography (HPLC), Polyacrylamide Gel Electrophoresis (PAGE), and LC-MS, which showed that it mimicked native protein-DNA complexes [30]. Subsequently, the characterized complex was point-labeled with bromo- derivative of nucleobases, irradiated (with X-rays or photons with 280 and 320 nm wavelength), and the resulting radio- and photo-damage was analyzed by LC-MS and dHPLC.

### 2.2. X-ray Experiments

X-ray irradiated (700 Gy) dsDNA and dsDNA-PEP systems were analyzed by dHPLC and LC-MS methods. The dHPLC analysis allowed for estimation of the degree of DNA degradation after radiation. To calculate the degradation rate, the area of the ssDNA-A and ssDNA-B peaks were measured. The degradation rate of the material in relation to the control was obtained from the chromatograms contained in Figures S1 and S2 and are presented in Table 1. Data obtained by HPLC indicated that after X-ray radiation, the sensitized oligonucleotides (BrdA, BrdC, BrdG, BrdU) are more susceptible to damage than native DNA. The highest degree of degradation can be observed with BrdA labeled material (82 ± 4%, 61 ± 3% for dsDNA and dsDNA-PEP, respectively). Similarly, DNA damage was detected in BrdC labeled material (81 ± 4%, 73 ± 6% for dsDNA and dsDNA-PEP, respectively) and BrdU (76 ± 4%, 61 ± 3% for dsDNA and dsDNA-PEP, respectively) and significantly less damage was noted for BrdG labeled material (51 ± 1%, 57 ± 2% for dsDNA and dsDNA-PEP, respectively). The observed X-ray DNA damage products and yields are in accordance with the literature data [2,4,13,31].

In addition, only for the BrdG labeled DNA, the peptide-interacting system was more damaged than the peptide-free system. This is most likely related to the specific interaction of BrdG with the arginine amino acid residue, which significantly improves the electron-accepting properties of the G/ARG pair. For the other sensitizers, there is no direct contact with positively charged amino acid residues. For the BrdA, BrdC, and BrdU, less degradation was noted for DNA/peptide complexes. To explain this regularity, attention may be paid to strands that are not labeled with dsDNA and dsDNA-PEP sensitizers, where their degradation triggered by X-rays is 32 ± 2% and 21 ± 5%, respectively. However, the degradation of the complementary strand occurs at the level of approx. 25% in all cases. It can be concluded that about 25–30% of damage occurs independently of the presence of a sensitizer, where the presence increases the effect of X-rays (degradation of the labeled threads is further increased by ~45% except for BrdG—20% labeled strands).

**Figure 1:**
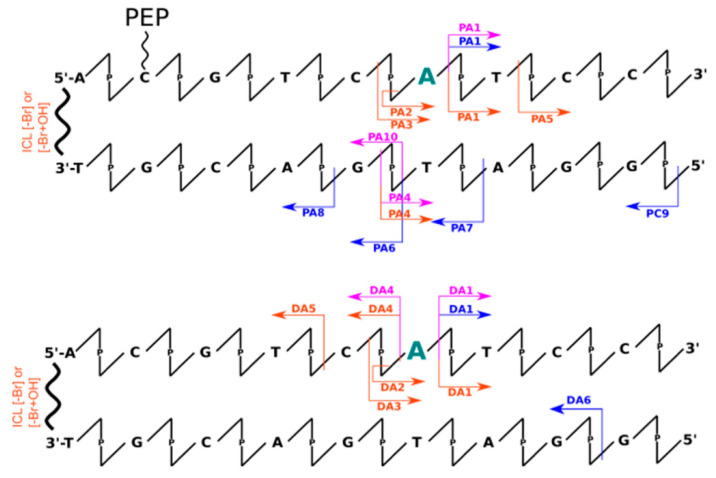
Schematic representation of all identified oligonucleotides’ fragments by means of LC-MS for BrdA labeled systems (upper with peptide conjugate, lower without peptide conjugation). The scheme represents only part of the dsDNA structure. The arrows represent the respective fragments identified for X-ray radiation experiments (blue), UV irradiation by 320 nm photons (pink), and 280 nm photons (orange). Modified nucleobase was pointed in green. Below the arrows the names of identified fragments are listed starting with “P” stands for system with peptide conjugation, starting with “D” without peptide conjugation. Additionally, the interstrands cross-links (ICL) were observed. For possible mechanisms, please see Figures S7–S11.

**Figure 2:**
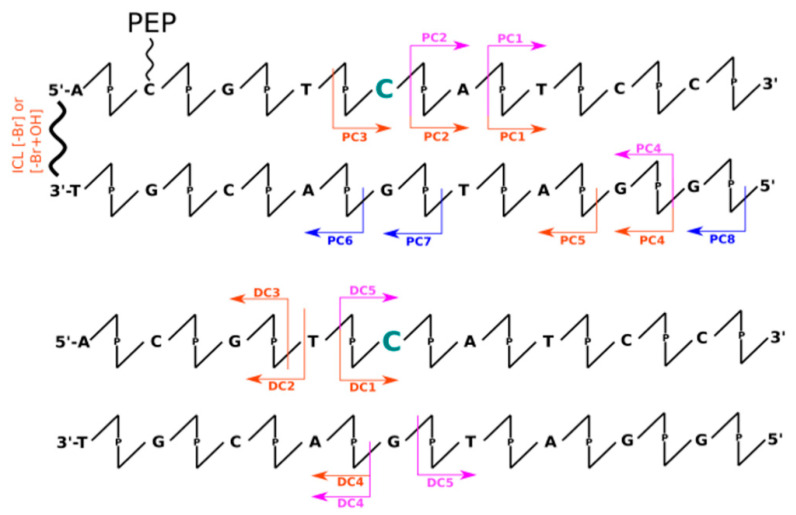
Schematic representation of all identified oligonucleotides’ fragments by means of LC-MS for BrdC labeled systems (upper with peptide conjugate, lower without peptide conjugation). The scheme represents only part of the dsDNA structure. The arrows represent the respective fragments identified for X-ray radiation experiments (blue), UV irradiation by 320 nm photons (pink), and 280 nm photons (orange). Modified nucleobase was pointed in green. Below the arrows the names of identified fragments are listed starting with “P” stands for system with peptide conjugation, starting with “D” without peptide conjugation. Additionally, the interstrands cross-links (ICL) were observed. For possible mechanisms please see Figures S7–S11.

**Figure 3:**
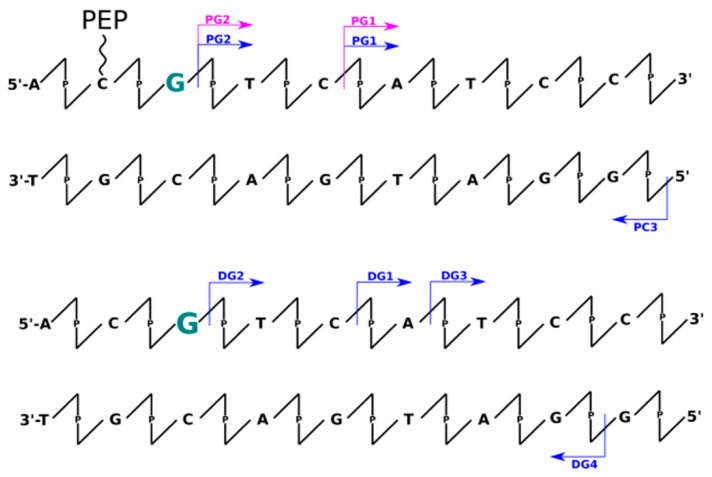
Schematic representation of all identified oligonucleotides’ fragments by means of LC-MS for BrdG labeled systems (upper with peptide conjugate, lower without peptide conjugation). The scheme represents only part of the dsDNA structure. The arrows represent the respective fragments identified for X-ray radiation experiments (blue), UV irradiation by 320 nm photons (pink), and 280 nm photons (orange). Modified nucleobase was pointed in green. Below the arrows the names of identified fragments are listed starting with “P” stands for system with peptide conjugation, starting with “D” without peptide conjugation. Additionally, the interstrands cross-links (ICL) were observed. For possible mechanisms, please see Figures S7–S11.

**Figure 4:**
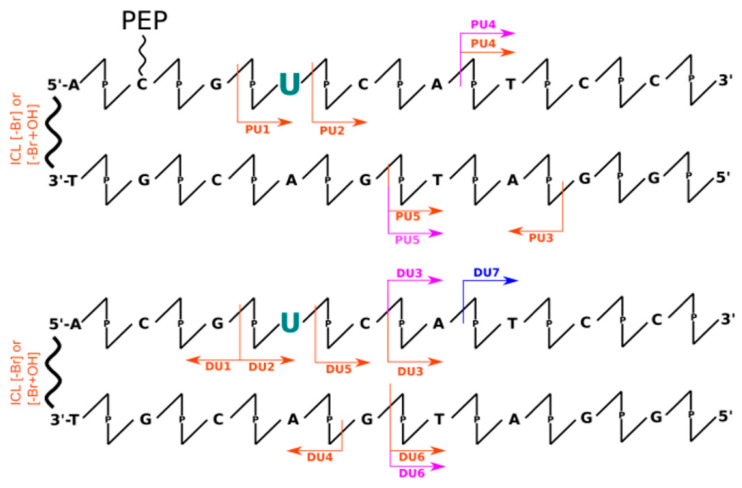
Schematic representation of all identified oligonucleotides’ fragments by means of LC-MS for BrdU labeled systems (upper with peptide conjugate, lower without peptide conjugation). The scheme represents only part of the dsDNA structure. The arrows represent the respective fragments identified for X-ray radiation experiments (blue), UV irradiation by 320 nm photons (pink), and 280 nm photons (orange). Modified nucleobase was pointed in green. Below the arrows the names of identified fragments are listed starting with “P” stands for system with peptide conjugation, starting with “D” without peptide conjugation. Additionally, the interstrands cross-links (ICL) were observed. For possible mechanisms, please see Figures S7–S11.

Analysis of data obtained the LC-MS technique allowed for insight into the mechanisms occurring during irradiation indirectly through the identification of individual fragments of DNA degradation. Therefore, when analyzing MS data (see Table S1), one can observe the formation of DNA strand fragments as well as systems with larger masses than the initial material. This can be directly observed in the chromatograms of the total ionic current (see Figures S3 and S4). Thus, we can distinguish all the smaller DNA fragments, that result from breaking phosphodiester bond along the DNA strand, by a lower retention time (<17 min) than the signals from intact oligonucleotides. In addition, products that elute immediately after the retention time of the undamaged strands can be distinguished based on the chromatographic peak elongating and become asymmetrical. A deeper analysis of the masses of these fragments (see Figures S5 and S6) allowed us to observe, only in irradiated material, masses greater by a multiple of ~16 Da (see Figures S5 and S6), which most likely corresponds to the attachment of an -OH group to the DNA molecule. It is worth noting here that during the experiments a scavenger of hydroxyl radicals (*t*-butanol) was used, although according to the LC-MS data, we can still observe adducts of hydroxyl radicals to DNA. This is likely due to the presence of these hydroxyl radicals in the first hydration layer of DNA, where *t*-butanol may have a limited effect due to the internal structure of the water molecules. This additionally confirms the analysis of the degree between systems with or without peptide. The presence of the peptide significantly reduces the degree of strand degradation, which involves the displacement of water molecules by the peptide due to binding of the α-helix within the large groove of DNA or just by the scavenging ability of the peptide. In addition, we can also interpret the role of the peptide as a specific hydroxyl radical scavenger, by positively charged amino acids. All chromatographic peaks between 1 and 17 min retention time (Figures S1–S4—blue line) correspond to possible fragments of oligonucleotides (labeled and not labeled with sensitizers), in which the phosphodiester bond was broken. This is most likely due to the presence of the above-mentioned hydroxyl radicals, which are capable of damaging the oligonucleotide (regarding the sensitizer properties). Unfortunately, due to the presence of these non-specific defects, identification of products with specific damage caused by the DNA sensitizer is impossible. In Figure 1, Figure 2, Figure 3 and Figure 4 the most abundant radiolysis products of the dsDNA-PEP and dsDNA labeled material were provided. Unfortunately, analysis of these fragments does not allow for determination of the mechanism of their creation and direct interpretation whether these fragments arose due to the sensitizing properties of the radiosensitizer.

### 2.3. UV Experiments

The analysis of data obtained using the dHPLC technique (see Figures S1 and S2) allowed for a quantitative analysis of the degree of degradation of individually labeled strands as well as non-labeled ones. Table 1 summarizes the quantitative degree of degradation by photolysis obtained by dHPLC analysis. From these data, it is clear that unlabeled systems are resistant (degradation rate ~5%) to UV radiation in the 280 nm and 320 nm range. However, in the case of labeled strands, we can observe significant differences in the degree of degradation. Within the 320 nm irradiated systems, we observed an increased rate of degradation only in two systems: BrdC (0%, 20% for dsDNA and dsDNA-PEP, respectively) and BrdU labeled systems (0%, 20% for dsDNA and dsDNA-PEP) where damage was observed only in DNA/peptide complexes. In all other cases, the degree of DNA degradation was much lower ~5%, thus we conclude that the interaction of peptide is crucial for degradation. It is clear (see Table 1), that the material labeled with sensitizers is nearly insensitive to radiation in the 320 nm range, except for the abovementioned BrdU and BrdC in the dsDNA-PEP systems. As shown in Figure 1, Figure 2, Figure 3 and Figure 4, not all detected photolysis products correspond to the mechanisms of photoinduced degradation of DNA sensitized by BrdU described in the literature [15,16,17,22,31,32,33]—please find mechanism indicated in the Supplementary Materials (Figures S7–S11). We should not observe the DNA, or observe it in low yield. DNA damage of BrdU upon UV radiation should be highly sequence dependent [13], the BrdU molecule should be separated from guanine by adenines moieties in the same strand involving photo-induced electron transfer from G to BrdU and then lead do DNA damage [32]. The degree of degradation is also influenced by the process associated with the debromation of sensitizers (masses resulting from the replacement of the bromine atom by hydrogen have been observed). The resulting products after 280 nm photon exposure, in some cases, corresponded to the fragments that were expected from the process of PET as previously described in the literature. The degree of degradation of individual labeled strands: BrdA (51 ± 2%, 53 ± 2% for dsDNA and dsDNA-PEP, respectively), BrdG (8 ± 2%, 14 ± 2% for dsDNA and dsDNA-PEP, respectively), BrdC (24 ± 3%, 32 ± 2% for dsDNA and dsDNA-PEP, respectively), BrdU (6 ± 1%, 29 ± 1% for dsDNA and dsDNA-PEP, respectively), shows that, as with X-rays, the BrdG labeled material is the least sensitive to radiation. In addition, the rate of degradation is much higher for labeled DNA strands.

#### 2.3.1. UV Experiments—BrdA Labeled System

Interestingly, in the case of non-labeled dsDNA and dsDNA-PEP there is very little damage. Fragments identified by LC-MS suggest that in the case of BrdA labeled material, the processes described by Watanabe et al. [22] occurred, resulting in the elimination of the nucleoside towards 5′- and the β-elimination process (see Figure S9). Identified fragments DA3, DA5, PA1 (Figure 1) suggest the occurrence of photoinduced electron transfer (PET), while the fragments DA2 and PA2 indicate the occurrence of the β-elimination process (see Figure S9), whose final products have been observed. However, the remaining fragments recognized for BrdA labeled systems do not correspond to the theory of electron transfer, and are most likely related to the migration of charge, because photolesions can be observed in the guanine region (on both the labeled and complementary strand in vicinity of sensitizer). In addition, masses corresponding to a cross-link between strands was observed and such damage is among the most lethal to the cell. It can be clearly observed that the sensitizer takes a direct part in the formation of cross-links, because the molecular mass of the resulting conjugate was reduced by the mass of the bromine atom.

#### 2.3.2. UV Experiments—BrdC Labeled System

In the case of BrdC labeled systems, we can distinguish fragments that suggest PET [22] occurrence (DC1, DC3, PC3) and the elimination process in the 5′ direction of the labeled strand (Figure 2). However, it is difficult to explain the occurrence of other fragments for the dsDNA-PEP system. The remaining fragments suggest the existence of an additional mechanism that causes oligonucleotide damage only in dsDNA-PEP labeled systems. In the BrdC labeled system, the damage occurs in the direction of electron donor, as in the case of fragments PC4 and PC5. On the other hand, fragments recognized from the complementary strand for the labeled system without peptide suggesting elimination of guanosine from the complementary strand (DC4, DC5). The similar result presented here are also presented by Zdrowowicz et al. [33] Moreover, a cross-link was identified, implying the formation of an interstrand cross-link in dsDNA-PEP system.

#### 2.3.3. UV Experiments—BrdG Labeled System

For the BrdG labeled system, which as mentioned above, was characterized by a low degree of sensitivity to UV radiation, please see Figure 3. Those labeled systems where the only ones which did not exhibit interstrand cross-links, although debromination was observed—see chromatographic data (Figures S1–S4 about 18 min retention time). What is interesting is the debromination rate (at 280 nm) was higher for dsDNA system than for dsDNA-PEP. In addition, we can distinguish that the labeled system is more resistant to 320 nm photons than 280 nm ones. This may be related to the tautomeric equilibrium, which changes the absorption spectrum of BrdG [23]. It is also important to mention that there were almost no degradation products identified by LC-MS analysis which goes alongside with the low degradation rate of BrdG labeled material, indicating low sensitivity of BrdG sensitizer to UV radiation.

#### 2.3.4. UV Experiments—BrdU Labeled System

The last system subjected to the analysis was the BrdU labeled system. Within the LC-MS analysis, fragments (Figure 4) corresponding to the occurrence of the β-elimination (see Figure S9) process of the sugar of the adjacent nucleobase in the 5′ direction (PU1, DU1, DU2) and elimination of the hydrogen atom from the deoxyribose molecule of the sensitizer nucleotide (PU2, DU5) were identified—which stays with the agreement with mechanisms proposed in the literature [13,22,23]. However, as in the case of the BrdU molecule, we can recognize the fragments responsible for elimination of the guanosine nucleoside (PU5, PU3, DU4, DU6). Similarly to BrdA and BrdC labeled systems, interstrand cross-links were also identified.

## 3. Materials and Methods

Oligonucleotides were purchased at Metabion (Planegg, Germany) and purified by means of HPLC: ssDNA A: 5′-GCA CGT CAT CCG TATAG-3′, ssDNA A*: 5′-GCA XGT CAT CCG TATAG-3′ X=C8-Alkyne dC, ssDNA-BrdG: 5′-GCA XYT CAT CCG TATAG-3′ X=C8-Alkyne dC Y=8-bromo2′-deoxyguanosine, ssDNA-BrdU: 5′-GCA XGY CAT CCG TATAG-3′ X=C8-Alkyne dC Y=5-bromo2′-deoxyuridine, ssDNA-BrdC: 5′-GCA XGT YAT CCG TATAG-3′ X=C8-Alkyne dC Y=5-bromo2′-deoxycitidine, ssDNA-BrdA: 5′-GCA XGT CYT CCG TATAG-3′ X=C8-Alkyne dC Y=8-bromo2′-deoxyadenosine, ssDNA B: 5′-CTA TAC GGA TGA CGT GC-3′. Modified azide moiety peptide was purchased from GeneCust (Boynes, France)—PEP: H-ASP-PRO-ALA-ALA-LEU-LYS-ARG-ALA-ARG-ASN-THR-GLU-ALA-ALA-ARG-ARG-SER-ARG-ALA-ARG-LYS-GLY-GLY-LYS(N_3_)-NH2. For clarity, please refer to all abbreviations and sequences listed below in Table 2. Following reagents were used and purchased from Merck, Darmstadt, Germany: Ascorbic acid (anhydrous, ≥99.99%), CuSO_4_ (anhydrous, powder, ≥99.99%), aminoguanidine hydrochloride (≥98%), THPTA: Tris(3-hydroxypropyltriazolylmethyl)amine (≥95%), Tris Buffered Saline (pH 8, powder), Triethylamine GC grade, Acetic Acid LC-MS grade, Sodium cacodylate trihydrate > 98%, *t*-butanol anhydrous, ≥99.5%, Acetonitrile (LC-MS grade), Methanol (LC-MS grade), 1,1,1,3,3,3-Hexafluoro-2-propanol (HFIP, LC-MS grade), 1xPBS solution—pH 7.4. In all experiments ultrapure water was used (Darmstadt, Merck Millipore system, Germany).

### 3.1. CLICK Reaction Protocol

**Figure 5:**
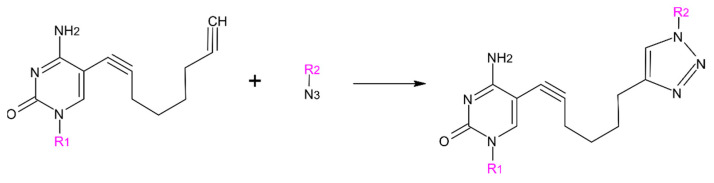
Illustration of the CLICK reaction enabling to link the oligonucleotide with peptide. R_1_—oligonucleotide, R_2_—peptide.

The CLICK reaction (Figure 5) was carried out in the same conditions as described previously [30]. In brief, the proper oligonucleotides (labeled by alkyne) were mixed with molar excess of peptide (labeled by azide) after stirring the mixture for ~1 h with freshly prepared ascorbic acid and t-butanol solutions the product was HPLC purified.

### 3.2. Annealing Protocol

Annealing was performed using an Eppendorf thermocycler (Eppendorf, Hamburg, Germany); equal amounts of lyophilized oligonucleotides or conjugate were resuspended in 1× PBS, mixed in 0.5 mL Eppendorf tubes to a final concentration of 10 μM. The temperature program was as follows: 5 min at 80 °C, 5 min at 55 °C, 60 min at 4 °C. The annealed product was observed by means of HPLC.X-ray experiment. Fifty microliter aliquots were irradiated in quartz capillary tubes (3 mm × 3 mm × 50 mm) in an X-ray Chamber (Faxitron, Tuscon, AZ, USA). The solutions were deoxygenated using argon (99.99999% purity). The dose was 700 Gy, and the exposure rate was 6.7 Gy/min at room temperature. *t*-butanol was used as a hydroxyl radical scavenger at final concentration of 10 mM).

### 3.3. UV Experiment

Exposure of dsDNA/dsDNA-PEP systems was performed in quartz capillaries (3 mm × 3 mm × 50 mm) with 50 μL of solution. The solutions were deoxygenated using argon (99.99999% purity). Exposure was carried out at two wavelengths: 280 nm (60 min, dose ~136 kJ/m^2^) and 320 nm (30 min, dose ~68 kJ/m^2^) using an optical table (OPTEL, Opole, Poland) equipped with a 500-watt mercury lamp (OSRAM, Munich, Germany) and monochromator (OPTEL, model M250, 2.5 nm width). The light intensity was approximately 38 Wm^−^^2^. Exposure was repeated twice in independent experiments. The radiation dose was estimated using a Maestro11 digital actinometer (Standa, Vilnius, Lithuania).

### 3.4. HPLC

The HPLC and denaturing HPLC (dHPLC) separation was carried out using the XBridge OST with a RP C18 column (2.5 µm in particle size, 4.6 × 50 mm) (Waters, Milford, MA, USA) and phase A: 50 mM TEAA (triethylamine acetate) and 1% Acetonitrile (ACN) (*v*/*v*) in water, B: 80% ACN (*v*/*v*) in water. For purification (before radiation experiment) and to check materials, a gradient of 0–20% Phase B at 25 °C was used at a 1 mL/min flow rate. Analysis of material after radiation was performed at the same condition but at an elevated temperature of 80 °C (dHPLC) to ensure the DNA melted into single strands. Separation was performed on a DionexUltiMate 3000 System (Thermo Fisher, Waltham, MA, USA) with Diode Array Detector (Thermo Fisher, Waltham, MA, USA) monitoring at 260 nm wavelength.

### 3.5. LC/MS

LC-MS analysis of DNA fragments was carried out using the ultra high-performance liquid chromatography (UPLC) system Nexera X2 (Shimadzu, Duisburg, Germany) coupled to a mass spectrometer TripleTOF 5600+ (AB SCIEX, Concord, ON, Canada) equipped with a duo-electrospray interface operated in negative ionizing mode. An acquity UPLC BEH C18 1.0 × 50 mm column (Waters) with a flow rate maintained at 0.1 mL/min was used for chromatographic separation of oligonucleotides and conjugates. The temperature of separation was maintained at 60 °C in order to assure the best separation for the undamaged DNA strands and the respective fragments after X-ray or UV exposure. The samples were desalted in situ on the column by diverting effluent to waste for 1 min after injection. The mobile phase A consisted of 400 mM HFIP (1,1,1,3,3,3-Hexafluoro-2-propanol) and 15 mM TEA (Triethylamine) in deionized ultrapure water, and mobile phase B consisted of 200 mM HFIP, 7.5 mM TEA 50% (*v*/*v*) MeOH, and 50% (*v*/*v*) H_2_O. MS operation parameters for negative ionizing mode were as follows: the spray voltage −4.5 kV, the nebulizer gas (N_2_) pressure 30 psi, collision energy at −10 V, declustering potential −150 V and the source temperature was maintained at 300 °C. The analysis was performed with 10 μL solutions of photo- or radiolites.

## 4. Conclusions

More and more people are diagnosed with cancer each year. To prevent cancer deaths, many research groups are searching for “cancer drugs”, both theoretically and experimentally [10,13,18]. Many new branches and directions of research have been created, including the study of the sensitization of DNA to radiation, because the majority of patients with localized cancers undergo radiation therapy and, less frequently, dynamic phototherapy [13]. The results obtained from this project should provide a deeper understanding of the radio- and photo-sensitization processes and allows for more purposeful design of new drugs. In summary, the damage resulting from X-ray radiation to sensitizer labeled and native DNA was mainly caused by hydroxyl radical species. Even when a radical scavenger was used in huge molar excess (*t*-butanol, 1000 molar excess), we identified DNA damage made by hydroxyl radicals, especially hydroxyl adducts and strand cleavage (H4′ hydrogen abstraction). These non-scavengeable hydroxyl radicals are probably generated in the first hydration sphere of DNA, which means that structured water molecules around DNA molecule are substrates for hydroxyl radical production. We can also observe that the amount of labeled strand after irradiation is significantly decreased in comparison with complementary strand which stand with the data presented in the literature [3,24,33]. Likely, we are observing the effect resulting from the DEA process, however the amount of debrominated strands is low. Interactions of the BrdG base with arginine significantly improves the electron affinity of this pair, causing an increase in the degree of DNA degradation under the influence of X-rays, which generates solvated electrons. The analysis of dsDNA systems labeled with sensitizers (BrdA, BrdG, BrdC, BrdU) showed an increase in the degree of DNA degradation in systems containing a sensitizer compared native DNA. In this case, the protective role of the peptide was observed, which displaced the water molecules from the vicinity of the large groove of DNA, by specifically interacting with it, and thereby reducing the number of non-specific lesions [4,26,27,28]. One of the most interesting processes analyzed during this work was the photochemical damage of sensitized oligonucleotides. It was possible to observe the influence of the peptide, which increased the degree of degradation of the sensitized DNA as well as the formation of extremely lethal interstrand cross-links. We observed previously described mechanisms related to photoinduced electron transfer leading to DNA degradation, as well as what are most likely previously unreported mechanisms responsible for photoinduced DNA damage which need further study to fully understand the mechanism leading to degradation.

To sum up, the susceptibility of photochemical or radiation DNA/Protein damage was investigated. Interactions of the BrdG base with the rest of arginine improve the electron affinity of this pair, lowering the rate of DNA degradation under X-ray radiation that generates solvated electrons. Thanks to the use of DNA sensitizers (BrdA, BrdG, BrdC, BrdU) we have observed increasing degradation rate of sensitized material in respect to the non-labeled DNA. Occurrence of nonspecific DNA damage was observed due to the formation of the non-scavengeable hydroxyl radicals, despite the use of a radical scavenger. In this case, the protective role of the peptide was evident. UV-light experiments clearly shows the effect of a interacting peptide, that increased the rate of degradation of the sensitized DNA and formation of interstarnd-cross links that are extremely lethal for cells.

## Figures and Tables

**Table 1:** Degradation rate [%] of all analyzed systems by means of HPLC—X-ray, UV (280 and 320 nm). Strand labeled “B” corresponds to the ssDNA B (see Materials and Methods) and is the same for all of the double stranded DNA molecules. Strand “L” corresponds to the ssDNA A or ssDNA A* for dsDNA- and dsDNA-PEP, repectively (see Materials and Methods). Strand “M *” corresponds to ssDNA-BrdA, ssDNA-BrdC, ssDNA-BrdG, ssDNA-BrdU respectively, with or without conjugation (dsDNA vs dsDNA-PEP). The degradation rate is the % of peak area of material which was exposed to radiation to material which was not exposed to radiation.

	**BrdA**						**BrdG**					
	**700 Gy**		**280 nm**		**320 nm**		**700 Gy**		**280 nm**		**320 nm**	
STRAND	M *	B	M *	B	M *	B	M *	B	M *	B	M *	B
dsDNA [%]	28 ± 1	82 ± 4	3 ± 1	51 ± 2	−2 ± 2	1 ± 2	14 ± 3	51 ± 1	6 ± 2	8 ± 2	−1 ± 2	4 ± 1
dsDNA-PEP [%]	31 ± 2	61 ± 3	14 ± 3	53 ± 2	1 ± 1	6 ± 4	30 ± 5	57 ± 2	12 ± 3	14 ± 2	2 ± 1	7 ± 1
	**BrdC**						**BrdU**					
	**700 Gy**		**280 nm**		**320 nm**		**700 Gy**		**280 nm**		**320 nm**	
STRAND	M *	B	M *	B	M *	B	M *	B	M *	B	M *	B
dsDNA [%]	26 ± 3	81 ± 4	4 ± 1	24 ± 3	−1 ± 2	0 ± 1	23 ± 1	76 ± 4	4 ± 1	6 ± 1	−3 ± 2	−1 ± 1
dsDNA-PEP [%]	35 ± 2	73 ± 6	15 ± 4	32 ± 2	17 ± 3	20 ± 5	33 ± 3	61 ± 3	15 ± 4	29 ± 1	7 ± 3	20 ± 2
	**Non-label material**											
	**700 Gy**		**280 nm**		**320 nm**							
STRAND	L	B	L	B	L	B						
dsDNA [%]	23 ± 3	32 ± 2	−3 ± 4	8 ± 2	1 ± 1	0 ± 1						
dsDNA-PEP [%]	19 ± 1	25 ± 5	1 ± 3	4 ± 3	1 ± 2	0 ± 1						

**Table 2:** The list of the oligonucleotides and peptide sequences with their respective abbreviations.

Name	Sequence
ssDNA A	5′-GCA CGT CAT CCG TATAG-3′
ssDNA A*	5′-GCA XGT CAT CCG TATAG-3′ (X=C8-Alkyne dC)
ssDNA-BrdG	5′-GCA XYT CAT CCG TATAG-3′ (X=C8-Alkyne dC Y=8-bromo2′-deoxyguanosine)
ssDNA-BrdU	5′-GCA XGY CAT CCG TATAG-3′ (X=C8-Alkyne dC Y=5-bromo2′-deoxyuridine)
ssDNA-BrdC	5′-GCA XGT YAT CCG TATAG-3′ (X=C8-Alkyne dC Y=5-bromo2′-deoxycitidine)
ssDNA-BrdA	5′-GCA XGT CYT CCG TATAG-3′ (X=C8-Alkyne dC Y=8-bromo2′-deoxyadenosine)
ssDNA B	5′-CTA TAC GGA TGA CGT GC-3′
PEP	H-ASP-PRO-ALA-ALA-LEU-LYS-ARG-ALA-ARG-ASN-THR-GLU-ALA-ALA-ARG-ARG-SER-ARG-ALA-ARG-LYS-GLY-GLY-LYS(N3)-NH2

## Supplementary Materials

Supplementary materials are available online. Figure S1. Absorbance chromatograms of labeled dsDNA after irradiation by X-ray, 320 nm or 280 nm wavelength; Figure S2. Absorbance chromatograms of labeled dsDNA-PEP after irradiation by X-ray, 320 nm or 280 nm wavelength; Figure S3. LC-MS chromatographic separation of labeled dsDNA after irradiation by X-ray, 320 nm or 280 nm wavelength; Figure S4. LC-MS chromatographic separation of labeled dsDNA-PEP after irradiation by X-ray, 320 nm or 280 nm wavelength; Figure S5. The ionic current that equates the hydroxyl adducts to one of DNA strands: material non-irradiated (pink color) and irradiated (color blue); Figure S6. Ion current deconvolution representing hydroxyl adducts to one of the DNA strands: non-irradiated material (pink color) and irradiated (blue); Table S1. Table represents the retention time and masses of fragments found during LC-MS analysis. Figure S7. Mechanism leading to a basic site formation in DNA—schematic representation; Figure S8. Mechanism leading to aldehyde formation in DNA—schematic representation; Figure S9. Mechanism leading to β-elimination process in DNA—schematic representation; Figure S10. Mechanism leading to cyclo-derivative formation in DNA—schematic representation; Figure S11. Mechanism leading to ketone derivative formation in DNA—schematic representation.

## Sample Availability

Not available.

## References

[1] Alizadeh E., Orlando T.M., Sanche L. (2015). Biomolecular damage induced by ionizing radiation: The direct and indirect effects of low-energy electrons on DNA. Annu. Rev. Phys. Chem..

[2] Sage E., Harrison L. (2011). Clustered DNA lesion repair in eukaryotes: Relevance to mutagenesis and cell survival. Mutat. Res. Fundam. Mol. Mech. Mutagen..

[3] Eccles L.J., O’Neill P., Lomax M.E. (2011). Delayed repair of radiation induced clustered DNA damage: Friend or foe?. Mutat. Res. Fundam. Mol. Mech. Mutagen..

[4] Spotheim-Maurizot M., Davídková M. (2011). Radiation damage to DNA in DNA-protein complexes. Mutat. Res. Fundam. Mol. Mech. Mutagen..

[5] Cadet J., Richard Wagner J. (2013). DNA base damage by reactive oxygen species, oxidizing agents, and UV radiation. Cold Spring Harb. Perspect. Biol..

[6] Steenken S. (1989). Purine Bases, Nucleosides, and Nucleotides: Aqueous Solution Redox Chemistry and Transformation Reactions of Their Radical Cations and e~ and OH Adducts. Chem. Rev..

[7] Alizadeh E., Sanche L. (2012). Precursors of solvated electrons in radiobiological physics and chemistry. Chem. Rev..

[8] Gauduel Y., Pommeret S., Migus A., Antonetti A. (1990). Some evidence of ultrafast H_2_O^+^-water molecule reaction in femtosecond photoionization of pure liquid water: Influence on geminate pair recombination dynamics. Chem. Phys..

[9] Le Caë S. (2011). Water Radiolysis: Influence of Oxide Surfaces on H_2_ Production under Ionizing Radiation. Water.

[10] Baldacchino G., Brun E., Denden I., Bouhadoun S., Roux R., Khodja H., Sicard-Roselli C. (2019). Importance of radiolytic reactions during high-LET irradiation modalities: LET effect, role of O_2_ and radiosensitization by nanoparticles. Cancer Nanotechnol..

[11] Liao D., Johnson R.S. (2007). Hypoxia: A key regulator of angiogenesis in cancer. Springer.

[12] Ward J.F. (1988). DNA Damage Produced by Ionizing Radiation in Mammalian Cells: Identities, Mechanisms of Formation, and Reparability. Prog. Nucleic Acid Res. Mol. Biol..

[13] Rak J., Chomicz L., Wiczk J., Westphal K., Zdrowowicz M., Wityk P., Zyndul M., Makurat S., Golon Ł. (2015). Mechanisms of Damage to DNA Labeled with Electrophilic Nucleobases Induced by Ionizing or UV Radiation. J. Phys. Chem. B.

[14] Cupellini L., Wityk P., Mennucci B., Rak J. (2019). Photoinduced electron transfer in 5-bromouracil labeled DNA. A contrathermodynamic mechanism revisited by electron transfer theories. Phys. Chem. Chem. Phys..

[15] Zeng Y., Wang Y. (2006). Sequence-dependent formation of intrastrand crosslink products from the UVB irradiation of duplex DNA containing a 5-bromo-2′-deoxyuridine or 5-bromo-2′-deoxycytidine. Nucleic Acids Res..

[16] Chen T., Cook G.P., Koppisch A.T., Greenberg M.M. (2000). Investigation of the origin of the sequence selectivity for the 5-halo- 2’-deoxyuridine sensitization of DNA to damage by UV-irradiation. J. Am. Chem. Soc..

[17] Yokoyama H., Mizutani R. (2014). Structural Biology of DNA (6-4) Photoproducts Formed by Ultraviolet Radiation and Interactions with Their Binding Proteins. Int. J. Mol. Sci.

[18] Berens P.J.T., Molinier J. (2020). Formation and recognition of uv-induced dna damage within genome complexity. Int. J. Mol. Sci..

[19] Brem R., Zhang X., Xu Y.Z., Karran P. (2015). UVA photoactivation of DNA containing halogenated thiopyrimidines induces cytotoxic DNA lesions. J. Photochem. Photobiol. B Biol..

[20] Cecchini S., Masson C., La Madeleine C., Huels M.A., Sanche L., Wagner J.R., Hunting D.J. (2005). Interstrand Cross-Link Induction by UV Radiation in Bromodeoxyuridine-Substituted DNA: Dependence on DNA Conformation. ACS Publ..

[21] Wieczór M., Wityk P., Czub J., Chomicz L., Rak J. (2014). A first-principles study of electron attachment to the fully hydrated bromonucleobases. Chem. Phys. Lett..

[22] Watanabe T., Tashiro R., Sugiyama H. (2007). Photoreaction at 5′-(G/C)AABrUT-3′ sequence in duplex DNA: Efficent generation of uracil-5-yl radical by charge transfer. J. Am. Chem. Soc..

[23] Wityk P., Zdrowowicz M., Wiczk J., Rak J. (2017). UV-induced electron transfer between triethylamine and 5-bromo-2′-deoxyuridine. A puzzle concerning the photochemical debromination of labeled DNA. J. Pharm. Biomed. Anal..

[24] Luscombe N.M., Laskowski R.A., Thornton J.M. (2001). Amino acid-base interactions: A three-dimensional analysis of protein-DNA interactions at an atomic level. Nucleic Acids Res..

[25] Rohs R., Jin X., West S.M., Joshi R., Honig B., Mann R.S. (2010). Origins of specificity in protein-DNA recognition. Annu. Rev. Biochem..

[26] Smith T.A., Kirkpatrick D.R., Smith S., Smith T.K., Pearson T., Kailasam A., Herrmann K.Z., Schubert J., Agrawal D.K. (2017). Radioprotective agents to prevent cellular damage due to ionizing radiation. J. Transl. Med..

[27] Brand M., Sommer M., Jermusek F., Fahl W.E., Uder M. (2018). Reduction of X-ray-induced DNA damage in normal human cells treated with the PrC-210 radioprotector. Biol. Open.

[28] Henderson R. (1990). Cryo-protection of protein crystals against radiation damage in electron and X-ray diffraction. Proc. R. Soc. B Biol. Sci..

[29] Wityk P., Wieczo M., Makurat S., Chomicz-Man L., Czub J., Rak J. (2017). Dominant Pathways of Adenosyl Radical-Induced DNA Damage Revealed by QM/MM Metadynamics. ACS Publ..

[30] Wityk P., Piątek R., Nowak R., Kostrzewa-Nowak D. (2020). Generation and characterization of a DNA-GCN4 oligonucleotide-peptide conjugate: The impact DNA/protein interactions on the sensitization of DNA. Molecules.

[31] Vogel S., Ebel K., Heck C., Schü R.M., Milosavljevic´ A.R., Milosavljevic´ M., Giuliani De A., Bald I. (1972). Vacuum-UV induced DNA strand breaks-influence of the radiosensitizers 5-bromouracil and 8-bromoadenine. Phys. Chem. Chem. Phys.

[32] Watanabe T., Bando T., Xu Y., Tashiro R., Sugiyama H. (2005). Efficient generation of 2′-deoxyuridin-5-yl at 5′-(G/C)AA xUxU-3′ (X = Br, I) sequences in duplex DNA under UV irradiation. J. Am. Chem. Soc..

[33] Zdrowowicz M., Wityk P., Michalska B., Rak J. (2016). 5-Bromo-2′-deoxycytidine—A potential DNA photosensitizer. Org. Biomol. Chem..

