# Supplementary material for: X-ray and UV Radiation Damage of dsDNA/Protein Complexes
Source: Molecules. 2021 May 24;26(11):3132. doi: 10.3390/molecules26113132 (PMC8197241; doi:10.3390/molecules26113132)
Supplement: Supplementary file 1 [file molecules-26-03132-s001.zip › molecules-1219561-supplementary.pdf]

X-ray and UV radiation damage of dsDNA/protein complexes

Paweł Wityk [1,2,3*], Dorota Kostrzewa-Nowak [4], Beata Krawczyk [1], Michał Michalik [5], Robert Nowak [4]

[1] Faculty of Chemistry, Gdańsk University of Technology, Narutowicza 11/12, 80-233 Gdańsk, Poland
[2] Faculty of Chemistry, University of Gdańsk, Wita Stwosza 63, 80-308 Gdańsk, Poland
[3] Department of Biopharmaceutics and Pharmacodynamics, Medical University of Gdańsk, Al. Gen. J. Halera 107, 80-416 Gdańsk, Poland
[4] Centre for Human Structural and Functional Research, University of Szczecin, 17C Narutowicza St., 70-240 Szczecin, Poland
[5] MML Medical Centre, Bagno 2, 00-112 Warsaw, Poland

* Correspondence: pawel.wityk@pg.edu.pl

ABSTRACT

Radiation and photodynamic therapies are used for cancer treatment by targeting DNA. However, efficiency is limited due to physio-chemical processes and the insensitivity of native nucleobases to damage. Thus, incorporation of radio- and photosensitizers into these therapies should increase both efficacy and the yield of DNA damage. To date, studies of sensitization processes have been performed on simple model systems e.g. buffered solutions of dsDNA or sensitizers alone. To fully understand the sensitization processes and to be able to develop new efficient sensitizers in the future, well established model systems are necessary. In the cell environment, DNA tightly interacts with proteins and incorporating this interaction is necessary to fully understand the DNA sensitization process. In this work, we used dsDNA/protein complexes labeled with photo- and radiosensitizers and investigated degradation pathways using LC-MS and HPLC after X-ray or UV radiation.

Fig. S1. Absorbance chromatograms of labeled dsDNA after irradiation by X-ray, 320 nm or 280 nm wavelength.

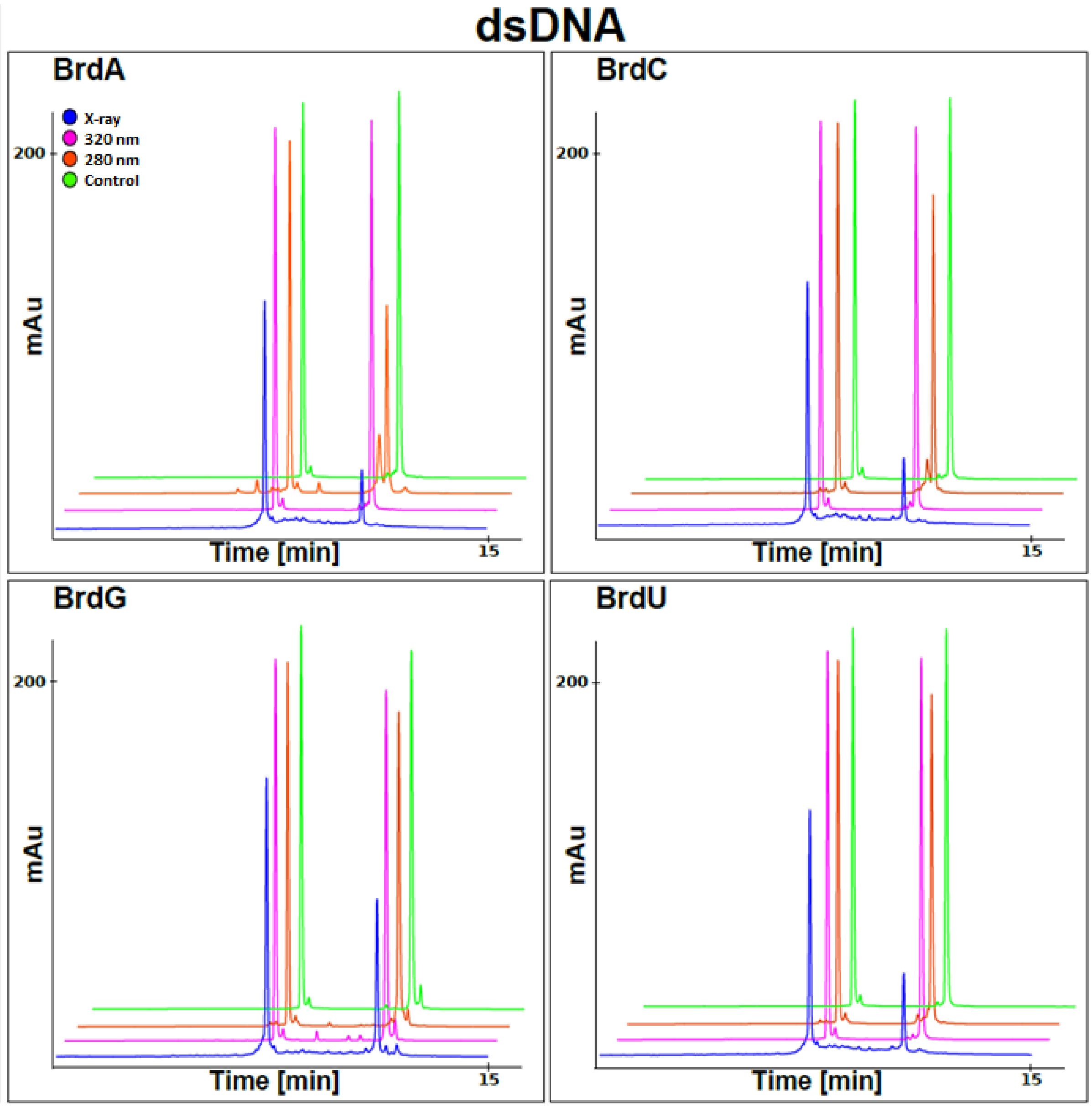

Fig. S2. Absorbance chromatograms of labeled dsDNA-PEP after irradiation by X-ray, 320 nm or 280 nm wavelength.

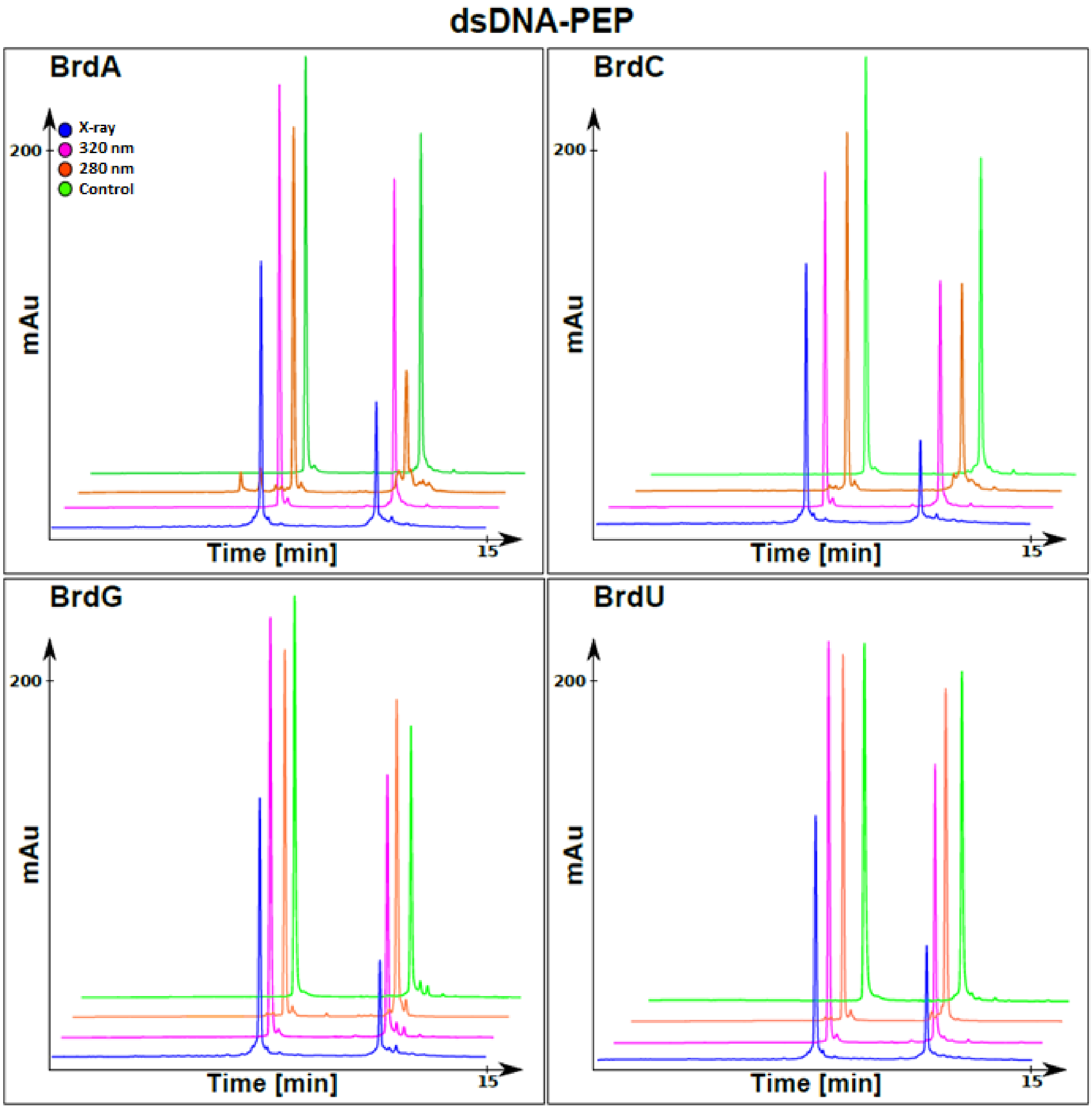

Fig. S3. LC-MS chromatographic separation of labeled dsDNA after irradiation by X-ray, 320 nm or 280 nm wavelength.

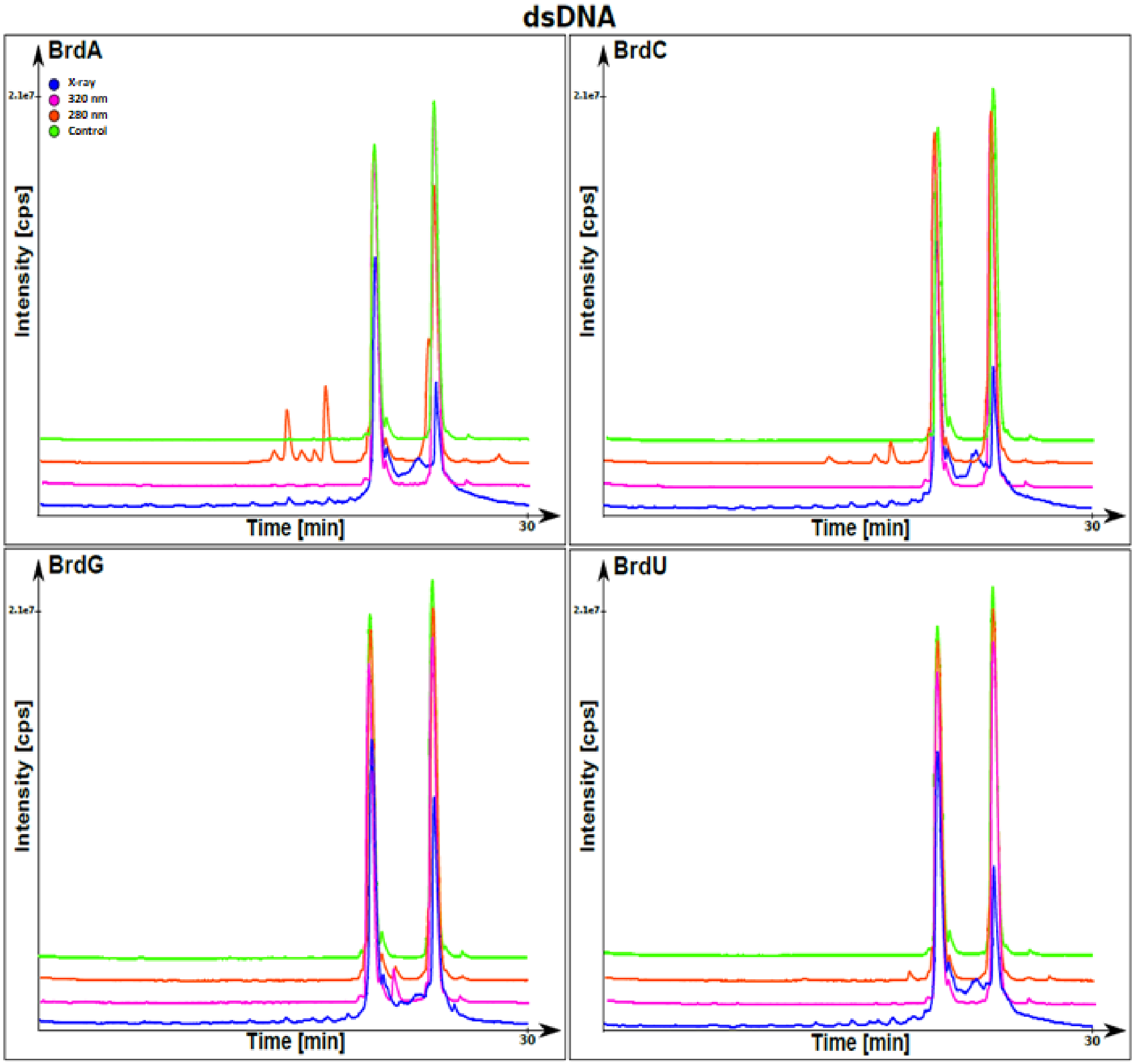

Fig S4. LC-MS chromatographic separation of labeled dsDNA-PEP after irradiation by X-ray, 320 nm or 280 nm wavelength.

dsDNA-PEP

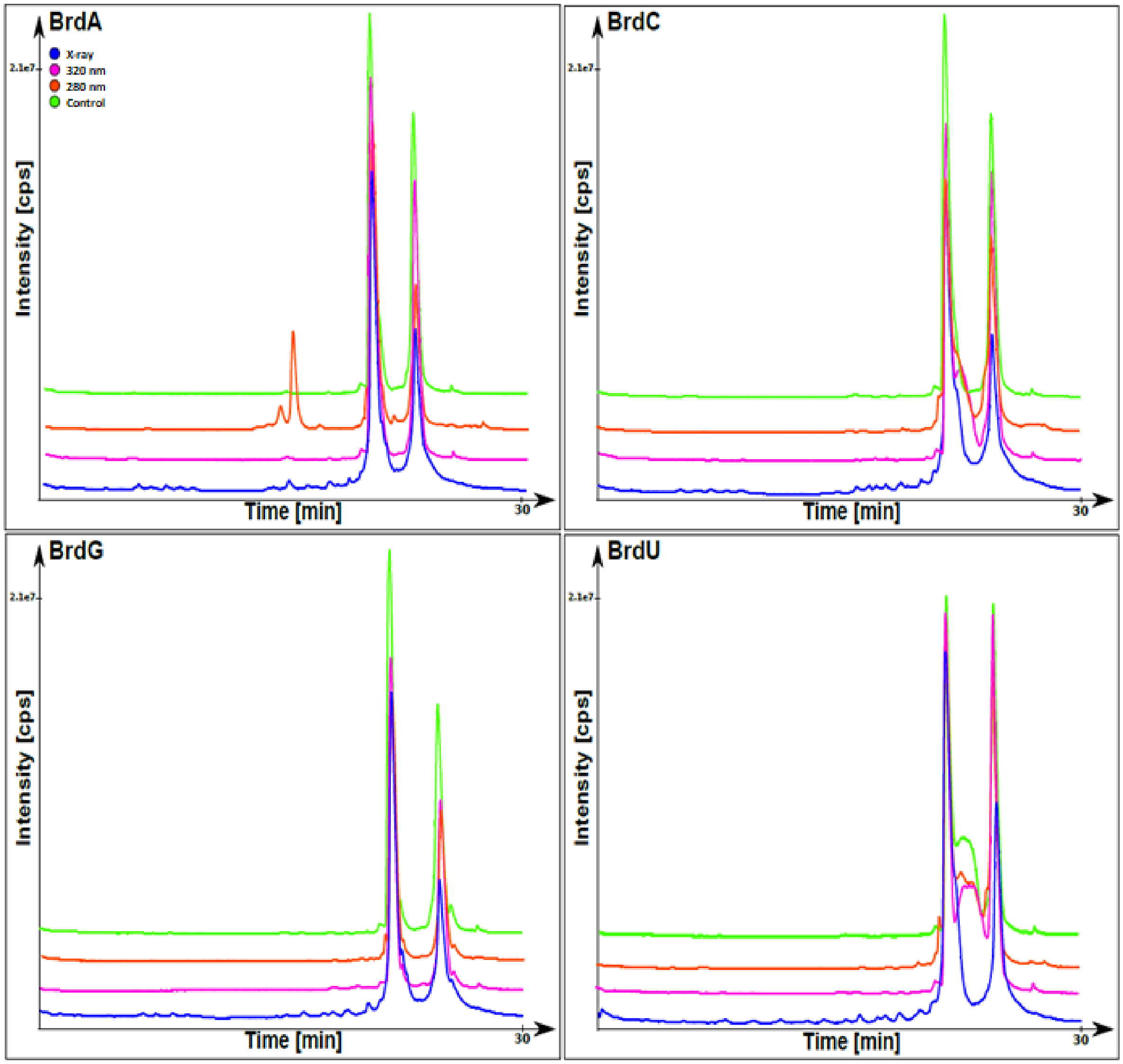
BrdA
X-ray
320 nm
280 nm
Control
2.1e7
Intensity [cps]
Time [min]
30
BrdC
2.1e7
Intensity [cps]
Time [min]
30
BrdG
2.1e7
Intensity [cps]
Time [min]
30
BrdU
2.1e7
Intensity [cps]
Time [min]
30

Fig. S5. The ionic current that equates the hydroxyl adducts to one of DNA strands: material non-irradiated (pink color) and irradiated (color blue).

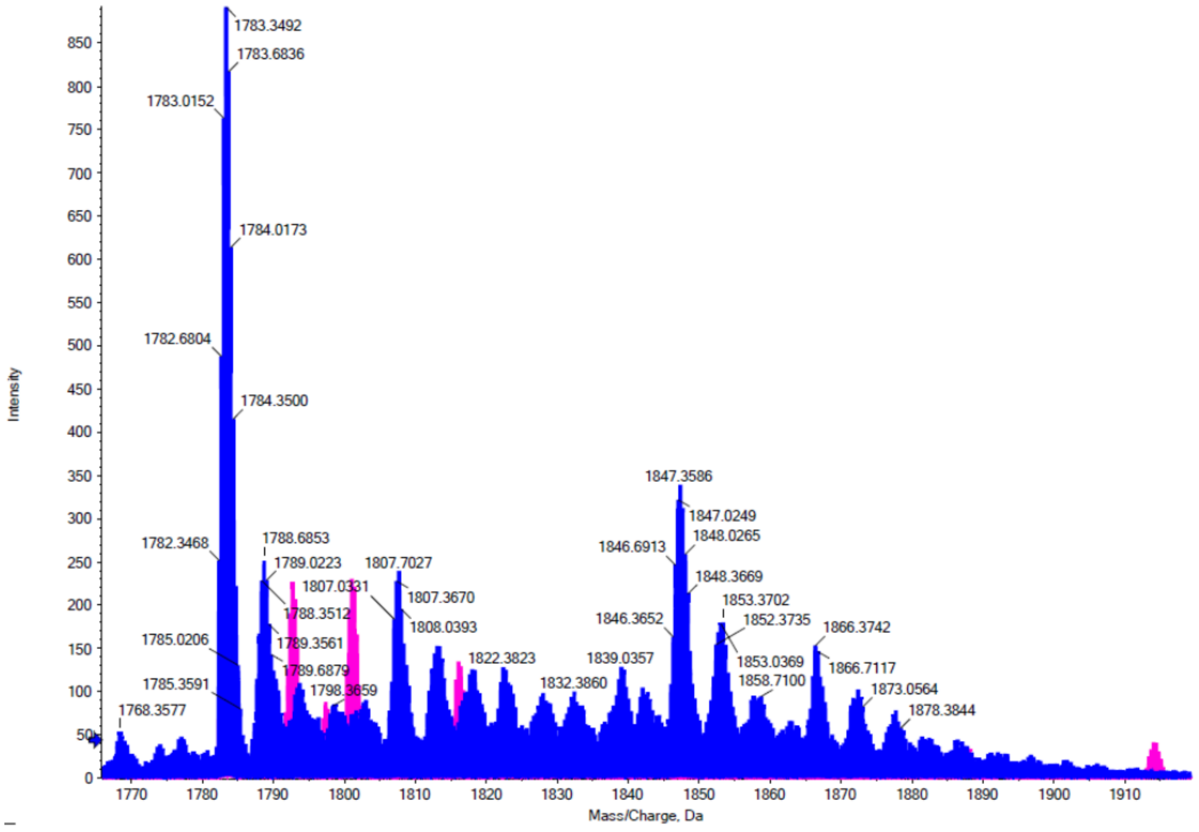

Fig S6. Ion current deconvolution representing hydroxyl adducts to one of the DNA strands: non-irradiated material (pink color) and irradiated (blue).

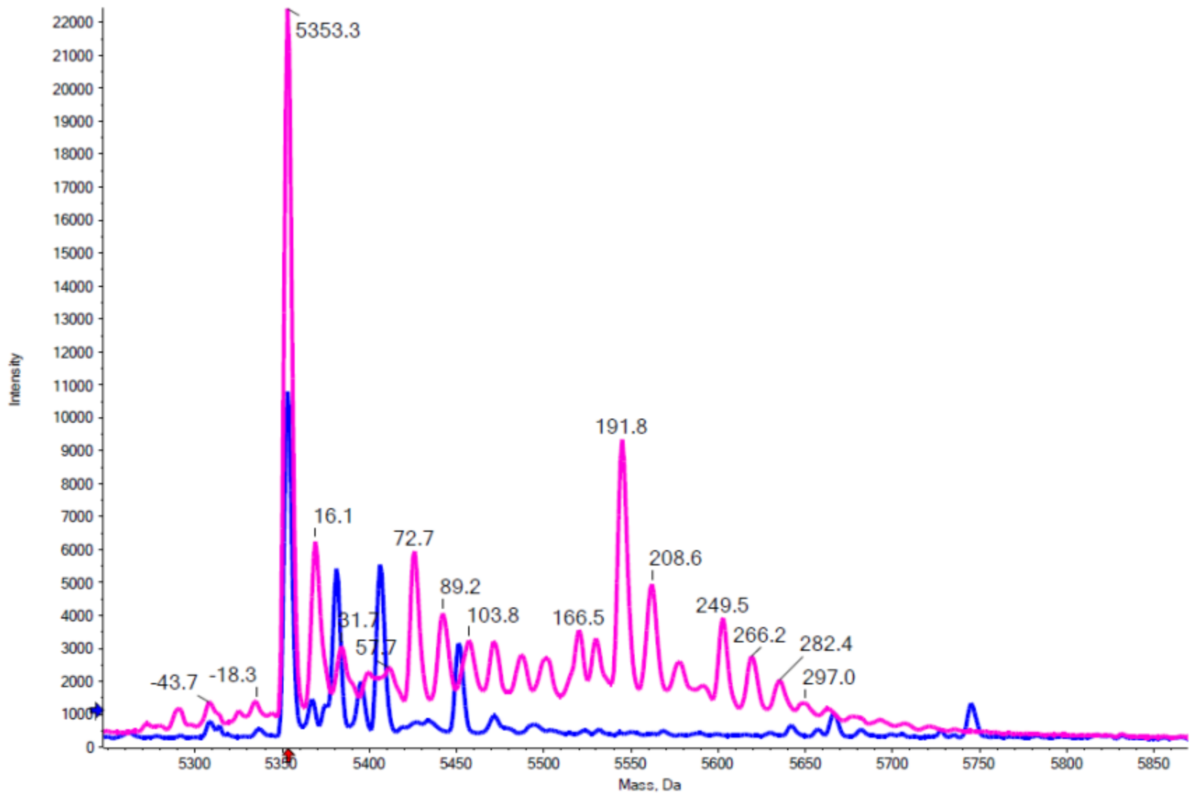

Table S1. Table represents the retention time and masses of fragments found during LC-MS analysis.

	Time [min]	Strand	Mass	Fragment
**1**	22.667	A	5170.940	cala
**2**	23.309	B	5210.940	cala
**3**	21.687	A	4527.696	b15
**4**	21.412	B	4591.722	b15
**5**	21.200	A	3700.341	w12
**6**	21.091	A	4223.584	b14
**7**	20.605	B	4287.643	b14
**8**	20.169	A	3396.265	w11
**9**	19.988	B	3766.366	w12
**10**	19.285	B	3669.416	b12
**11**	19.061	A	3606.384	b12
**12**	18.547	A	2754.003	w11-b15
**13**	18.115	A	2794.015	w9
**14**	17.933	B	3356.297	b11
**15**	17.933	A	3277.230	b11
**16**	16.775	B	2844.054	w12-b14
**17**	16.619	A	2988.157	b10
**18**	16.276	A	2488.909	w8
**19**	15.580	B	2529.921	w9-b16
**20**	15.250	B	2505.906	x8
**21**	14.909	B	2723.046	b9
**22**	14.317	A	2136.778	w14-b10/w13-b11
**23**	13.247	A/B	2200.808	w7/w14-b10;w9-b15
**24**	11.110	A	2394.921	b8
**25**	11.110	B	2394.921	w12-b12/w11-b13/w10-b14
**26**	10.322	A	1910.698	w6
**27**	10.322	B	1871.684	w6
**28**	9.511	B	2079.806	b7
**29**	8.865	A	1580.566	w5
**30**	8.154	B	1582.585	w6-b16/w7-b15/w8-b14/w12-b10/w14-b8/
**31**	7.950	B	1750.696	b6
**32**	7.950	B	1558.569	w5
**33**	7.613	A	1791.705	b6
**34**	6.861	A	1229.451	w14-b7/w9-b12/w8-b13
**35**	6.498	B	1462.595	b5
**36**	5.225	A	1488.602	b5
**37**	4.943	A/B	924.344	w-ACT-b
**38**	4.547	A/B	949.355	w-ACG-b
**39**	4.162	A/B	964.358	w-AGT-b
**40**	3.880	A/B	940.343	w-CGT-b
**41**	2.957	A/B	964.362	w-AGT-b
**42**	2.787	A/B	940.343	w-CGT-b
**43**	1.715	A/B	682.190	w-AC-c
**44**	1.7-0	A/B	All small fragments	

Fig. S7. Mechanism leading to abasic site formation in DNA– schematic representation.

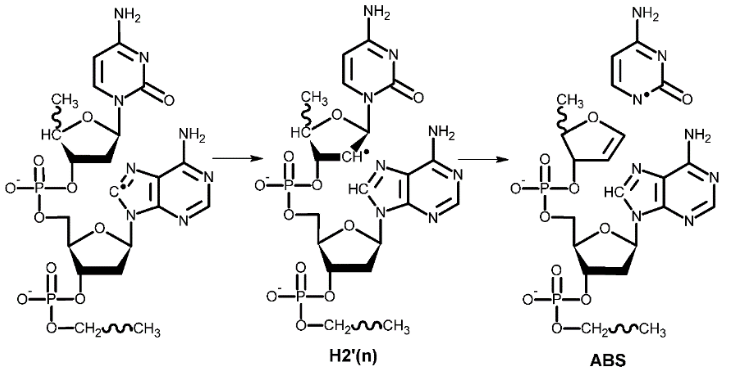

Fig. S8. Mechanism leading to aldehyde formation in DNA– schematic representation.

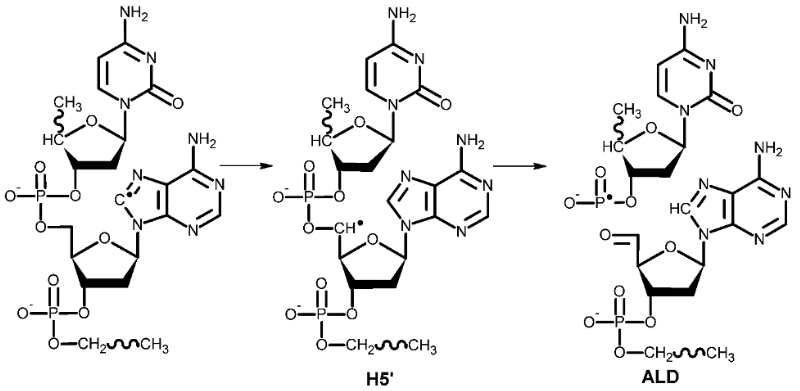

Fig. S9. Mechanism leading to β-elimination proces in DNA– schematic representation.

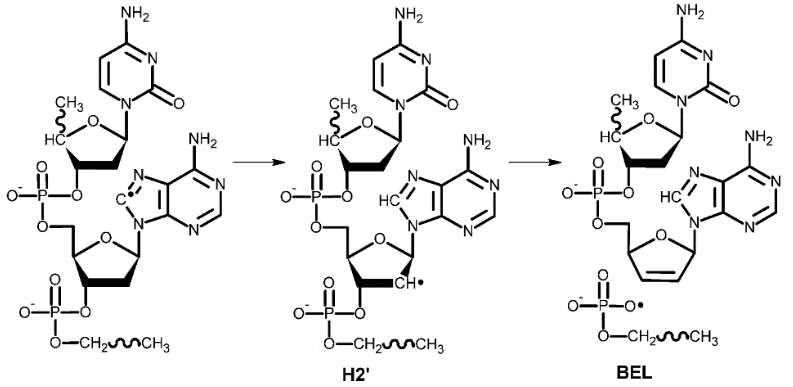

Fig. S10. Mechanism leading to cyclo-derivative formation in DNA– schematic representation.

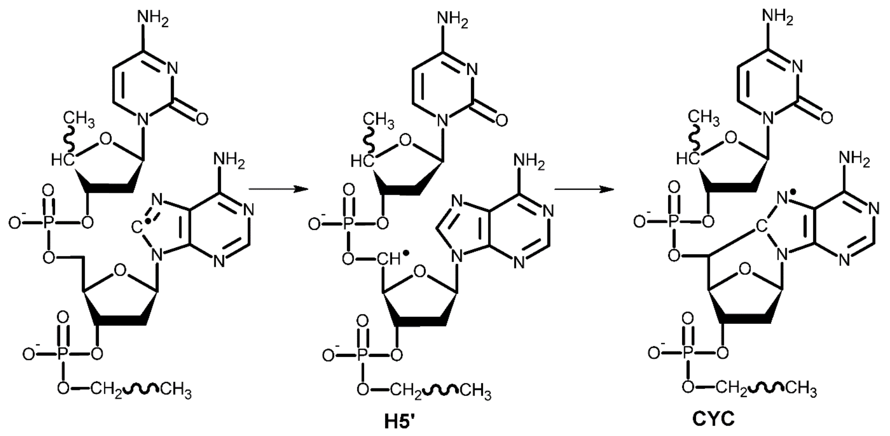

Fig. S11. Mechanism leading to ketone derivative formation in DNA– schematic representation.

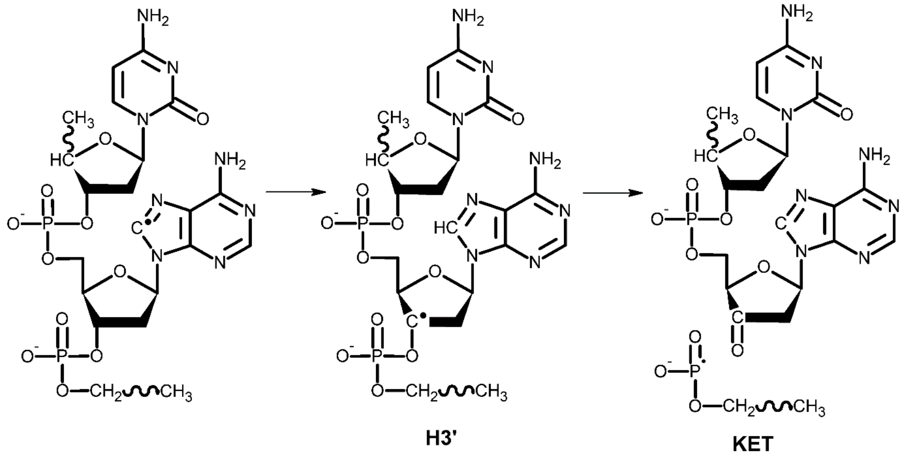

ACKNOWLEDGEMENT

This work was supported by the Polish Ministry of Science and Higher Education under the Grant No. 0138/DIA/2014/43 (P.W.). The calculations were supported by the PL-Grid Infrastructure (http://www.plgrid.pl/).The financial support of Polish Ministry of Science and Higher Education

(MNiSW) via Funds for Science and Polish Technology Programme that enabled the LC-MS system for the Nucleic Acid Laboratory at the Department of Chemistry University of Gdansk (Grant No. 775/FNiTP/127/2013 (J.R.) to be purchased is greatly acknowledged.
